# Primary melanoma of submandibular gland: case report and literature review of a very rare entity

**DOI:** 10.1186/s12903-022-02380-2

**Published:** 2022-08-14

**Authors:** Hassan Mir Mohammad Sadeghi, Ramtin Dastgir, Shaghayegh Bozorg Zadeh, Fatemeh Mashhadiabbas, Abbas Karimi, Meysam Mohammadi Khah

**Affiliations:** 1 Department of Oral and Maxillofacial Surgery, School of Dentistry, Shahid Beheshti University of Medical Sciences, Tehran, Iran; 2 Faculty of Dentistry, Tehran Medical Sciences, Islamic Azad University, Tehran, Iran; 3 Department of Oral and Maxillofacial Surgery, Taleghani Hospital, Mazandaran University of Medical Sciences, Chaloos, Mazandaran Iran; 4 Department of Oral and Maxillofacial Pathology, Dental School, Shahid Beheshti University of Medical Sciences, Tehran, Iran; 5 Department of Oral and Maxillofacial Surgery, Dental School, Tehran University of Medical Sciences, Tehran, Iran

**Keywords:** Primary melanoma, Neoplasm, Tumor, Submandibular gland, Case report

## Abstract

**Background:**

Cutaneous melanomas account for more than 95% of all cases of primary melanoma, making non-cutaneous primary melanomas truly rare. Cases of primary mucosal melanomas of the oral cavity have been widely described; however, instances of primary melanomas arising from salivary glands have been rarely described. To date, this is only the second case of primary melanoma of the submandibular gland.

**Case presentation:**

This is a report of a case of a 36-year-old healthy male patient, who was referred to us with the chief complaint of a growing swelling on the left side of his lower jaw. Evaluations revealed an evident facial asymmetry in the frontal view with a firm, non-tender swelling. Initial orthopantomogram did not reveal any alterations in the trabeculation or morphology of the jaws and the surrounding structures. A soft tissue ultrasonography of the left submandibular gland and anterior region of mandible revealed a hypoechoic cystic mass with numerous micro-echoes. Further para-clinical examinations yielded the definitive diagnosis of primary melanoma of the submandibular gland. Moreover, no evidence of distant osteometastasis was observed in whole-body scans. Subsequent surgical management with the approach of excising the submandibular salivary gland and concurrent selective neck dissection was implemented.

**Conclusions:**

This report emphasizes the importance of thorough examination and prompt referral to designated specialists in cases with suspicious behaviors which are unresponsive to treatments. It can be further concluded that melanoma can mimic a range of benign pathologies; therefore, putting it in the list differential diagnosis of similar lesions seems plausible.

## Background

Melanoma (also known as malignant melanoma [MM]) is a potentially aggressive and malignant tumor of uncontrolled replication of melanocytic cells; which are cells that are responsible for production and deposition of melanin pigment in the basal layer of epitheliums [1, 2]. Melanoma is the third most common cancer for men and the fifth most common for women in the United States, preceded by breast, prostate, colon, and uterine corpus cancer respectively; yet it only accounts for only 3–5% of all cutaneous cancers [1, 3]. In women between the ages of 25–35, melanoma comprises the leading cause of mortality arising from cancers. The most common age of occurrence is in the fifties to eighties, and it shows slightly higher prevalence in males; however, gender does not seem to affect the prognosis of the malignancy [4]. Melanoma is the most invasive form of skin cancer and according to the reports of the World Health Organization (WHO); mortalities arising from melanoma are progressing more rapidly than other types of cancer ever [5].

Cutaneous melanomas account for more than 95% of all cases of primary melanoma, therefore making non-cutaneous primary melanomas truly rare [6]. Cases of primary mucosal melanomas of the oral cavity have been widely described [7–9]; however, instances of primary melanomas originating from salivary glands have been very rarely described. To this day, this is only the second case of primary melanoma of the submandibular salivary gland. This report describes a case of a 36-year-old male diagnosed and treated for primary malignant melanoma of the submandibular gland.

## Case presentation

This is a report of a case of a 36-year-old healthy male, who was referred to the oral and maxillofacial surgery department of Taleghani hospital of Chaloos, Iran with the chief complaint of a growing swelling on the left side of his lower jaw. There was no history of underlying systemic conditions, trauma to the affected region, smoking, alcohol or substance abuse, and no hereditary diseases. The patient initially had presented to an outside dental clinic 4 months prior to being referred to us with the chief complaint of an extra-oral lesion measuring at 1 × 1 cm in the left submandibular region. Treatment with antibiotics and mouthwash rinses was erroneously prescribed by the dentist without further evaluating the etiology and nature of the lesion. Four months following the initial manifestations, the patient presented to an outside general surgeon’s clinic and an incisional biopsy of the lesion with an extraoral approach of the left submandibular region under local anesthesia was obtained. The pathology report confirmed the definitive diagnosis of malignant melanoma and therefore the patient was referred to the oral and maxillofacial department of Imam Hosein hospital of Tehran, Iran for further management.

### Physical examination

Upon the initial presentation, evaluations revealed an evident facial asymmetry in the frontal view with a firm, non-tender swelling measuring at approximately 5 × 4 cm in the left submandibular region, leaving the left inferior border of the mandible extra-orally non-palpable. A thorough and comprehensive examination of the whole-body skin and mucosa was undertaken based on the diagnosis of melanoma in order to identify potential sites of primary cutaneous melanomas, which were unavailing. Intraoral examination revealed a swelling measuring at 1 × 1 cm in the left mandibular distobuccal mucosa with no evidence of alterations in consistency, color, mucosal integrity, and tenderness on palpation. Furthermore, in intraoral examination, multiple patches of purple gingival discolorations measuring at 1 × 4 cm in dimensions were evident on the left side of the maxilla, expanding from the midline to the first premolar with no symptoms of pain or tenderness on palpation; however, the patient had not noticed this gingival discoloration previously and was unaware of its etiology and time of occurrence.

### Para-clinical tests

**Fig. 1:**
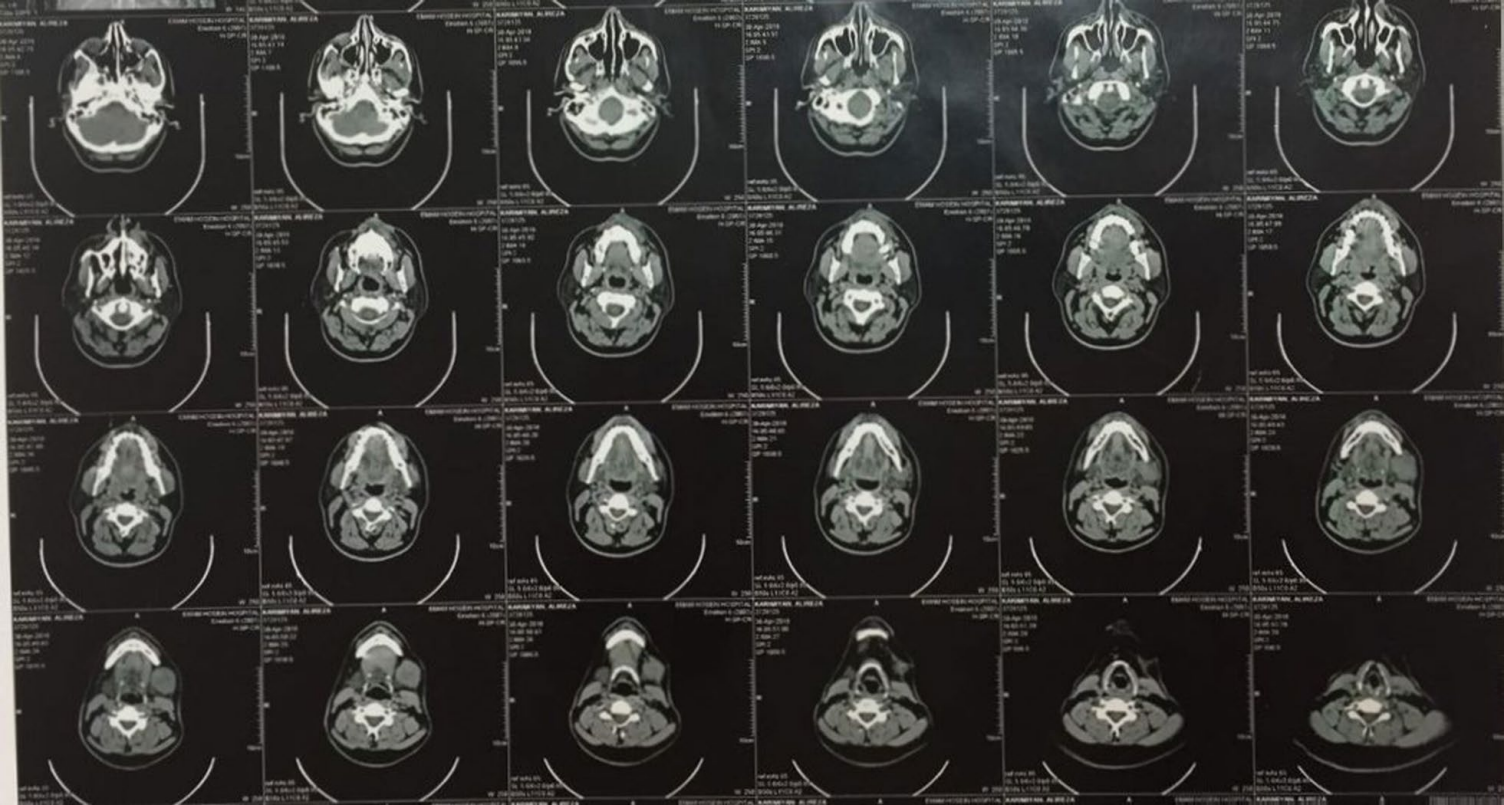
Cone beam computed tomography (CBCT) demonstrating soft tissue mass in the left submandibular gland and several oval shape cervical lymph nodes

**Fig. 2:**
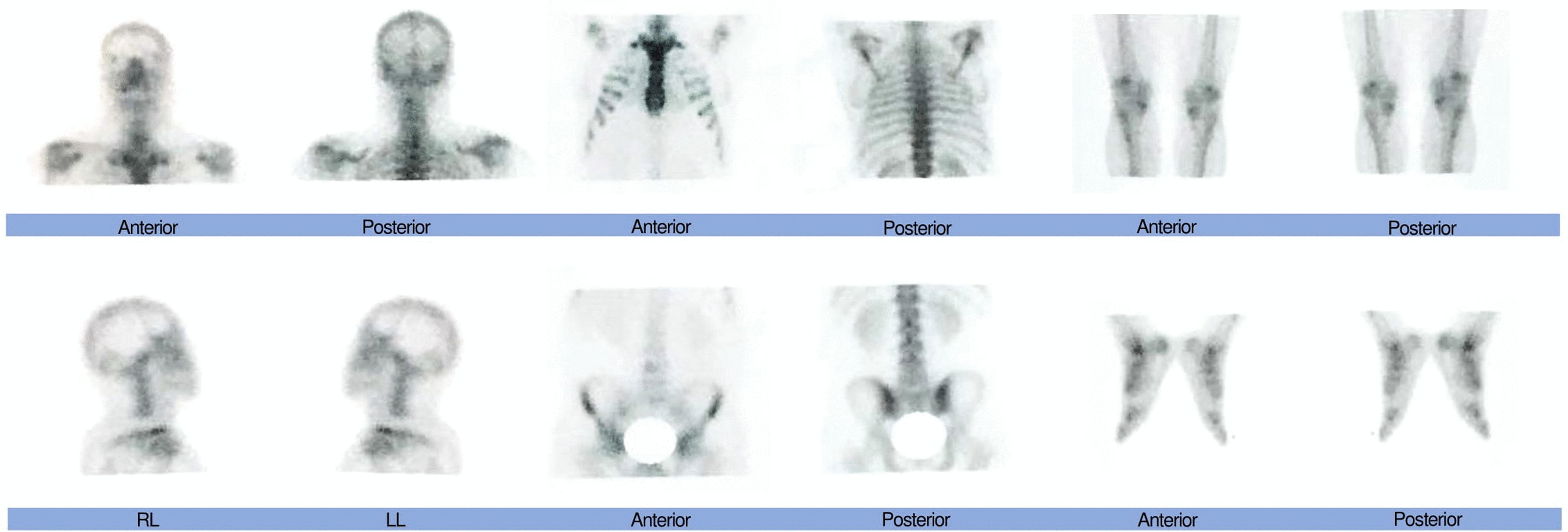
Whole-body bone scanning was preformed following IV injection of 740 MBq of Technetium 99 m-methyl diphosphonate (99mTc-MDP) both in anterior and posterior projections, showing no signs of distal metastasis

**Fig. 3:**
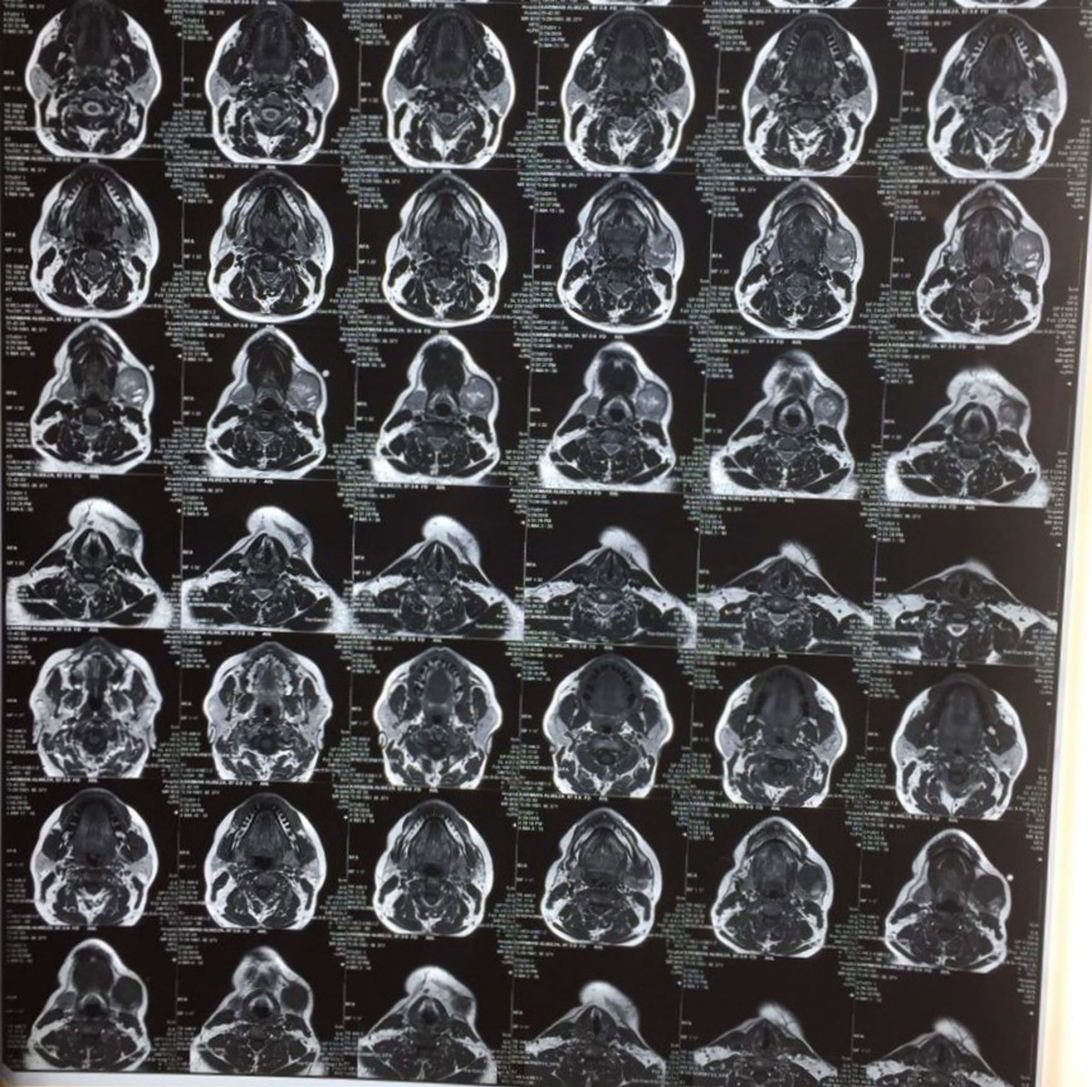
Sections of T1 Weighted MRI. There was evidence of a 28 × 24 mm large heterogenic mass in the left submandibular space

Initial orthopantomogram did not reveal any alterations in the trabeculation or normal anatomy and morphology of the jaws and the surrounding structures. A soft tissue ultrasonography of the left submandibular gland and anterior region of mandible was subsequently ordered which revealed a hypoechoic cystic mass with numerous micro-echoes with a diameter of 25 mm and a further septum were observed. Following these observations and the need for further cementing evidence, cone-beam computed tomography (CBCT) of the head and neck was ordered. In the evaluation of the CBCT, a soft tissue mass in the left submandibular area measuring at 41 × 33 mm and several oval shape cervical lymph nodes with maximum SAD: 5.5 mm were observed (Fig. 1). Whole-body bone scanning was preformed following IV injection of 740 MBq of Technetium 99 m-methyl diphosphonate (^99m^Tc-MDP) both in anterior and posterior projections. Normal distribution of radiotracer throughout the skeleton was observed and no significant osteopathology was detected. Therefore, it was concluded that no evidence of distant osteometastasis was evident in the whole-body scans (Fig. 2). Moreover, axial spiral chest and abdominopelvic CT-scans with contrast medium both demonstrated normal findings. In Magnetic Resonance Imaging (MRI) with and without contrast, on T1 Weighted view, evidence of a 28 × 24 mm mass was observed in the left submandibular space which can be attributed to lymphadenopathy (Fig. 3). Regarding the alteration in color of the aforementioned maxillary gingiva, an incisional biopsy was obtained in order to find whether there are any correlations between this lesion and the melanoma of the submandibular gland. Fortunately, no evidence of malignancy was observed in the histopathological evaluations and the lesion was definitively diagnosed as melanoacanthoma; therefore, ruling out the possibility of metastasis of the mandibular melanoma to the maxilla.

### Treatment plan

**Fig. 4:**
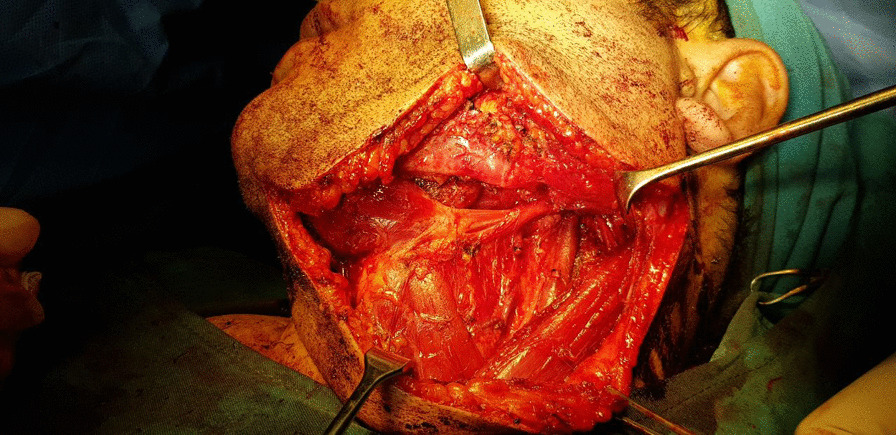
Following the dissection of the cervical cutaneous layers, undermining and sharp dissection of the platysma muscle was carried out in order to reach the superficial layer of the deep cervical fascia. The dissection was carried out through the deep cervical fascia and under the fascia to the inferior border of the mandible

**Fig. 5:**
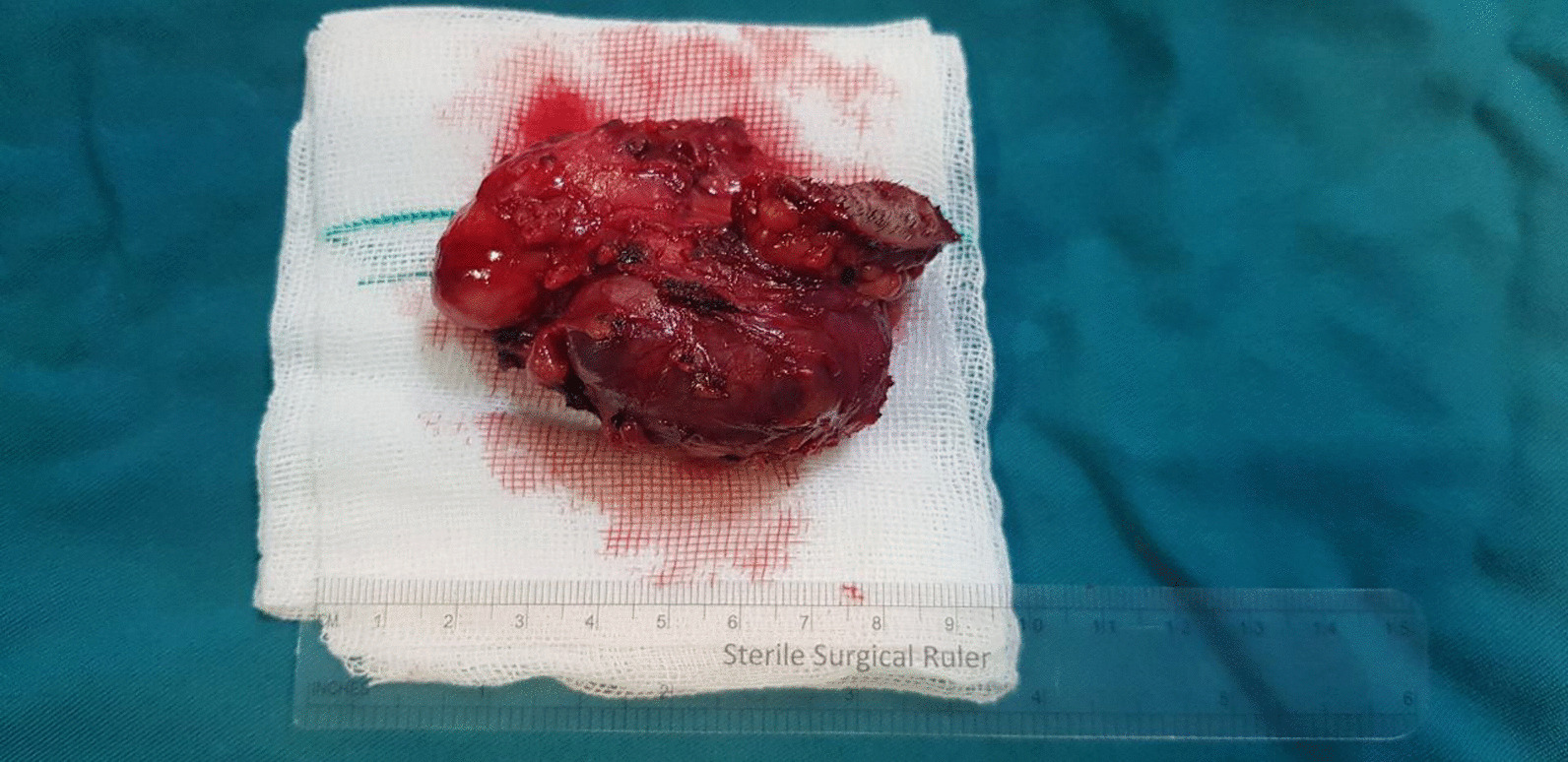
Excised submandibular salivary gland without any tear or rupture in its structure

The consensus of a surgical approach utilizing excision of the submandibular salivary gland and selective neck dissection under general anesthesia was reached. Following standard surgical prepping and draping for oral surgery, the incision was made by detecting margins of the lesion on skin taking into consideration a 1 cm safety margin. Local anesthesia was subsequently administered subcutaneously. Following the dissection of the cervical cutaneous layers, undermining and sharp dissection of the platysma muscle was undertaken in order to reach the superficial layer of the deep cervical fascia. The dissection was carried out through the deep cervical fascia and under the fascia to the inferior border of the mandible (Fig. 4). In this manner, the facial artery and vein and marginal mandibular branch of the facial nerve were detected and were subsequently preserved. The submandibular gland was spotted, suspensory elements incised, and the gland was excised without any tear or rupture in its structure (Fig. 5). Selective neck resection was undertaken through excision of lymph nodes of cervical levels IA, IB, IIA, IIB, III, and V. They were all then sent for frozen section biopsy analysis which all came back negative for metastasis. The excised submandibular gland was then preserved in formalin and sent for further pathological evaluations. The incised planes were subsequently reapproximated and sutured accordingly. The left maxillary oral melanoacanthoma didn’t need any surgical excision or treatment.

### Histopathological analysis

**Fig. 6:**
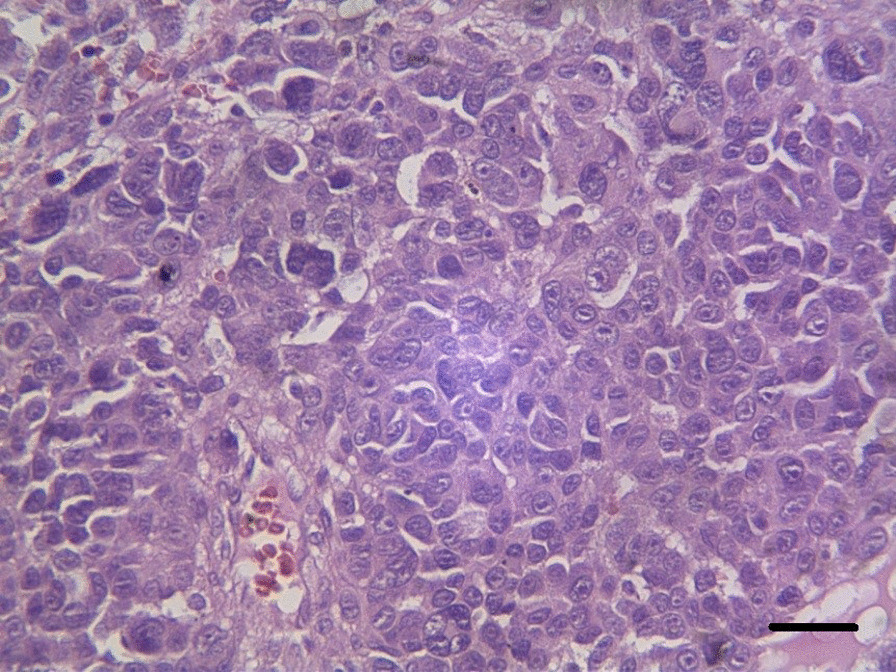
Histological section depicting lymphoid tissue involved by a malignant neoplasm composed of sheets of highly atypical cells with pleomorphic large vesicular nuclei, prominent nuclei, extensive necrosis, and the perivascular rosette (H&E staining, magnification 40 × 10, 30 μm scale bar, Olympus BX51 microscope, Nikon E400 camera)

**Fig. 7:**
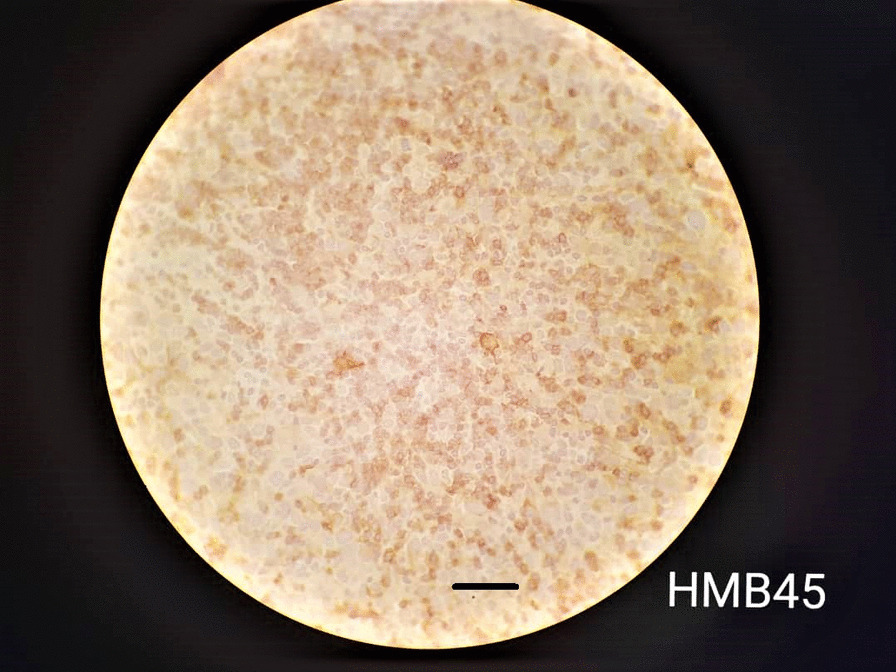
Immunohistochemical HMB45 staining yielding positive results and indicating melanoma (magnification 40 × 10, 30 μm scale bar, Olympus BX51 microscope, Nikon E400 camera)

**Fig. 8:**
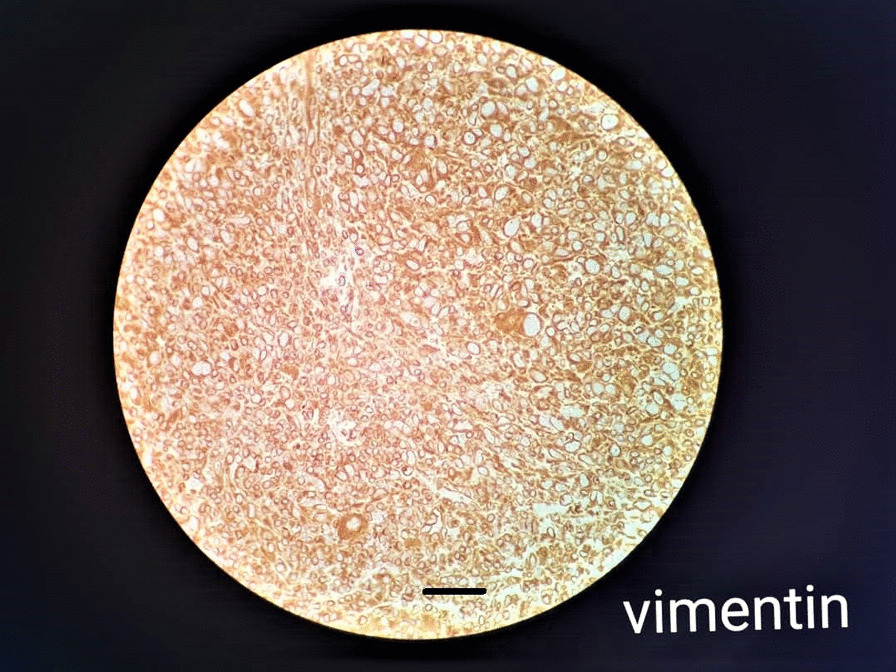
Immunohistochemical Vimentin staining yielding positive results and indicating melanoma (magnification 40 × 10, 30 μm scale bar, Olympus BX51 microscope, Nikon E400 camera)

**Fig. 9:**
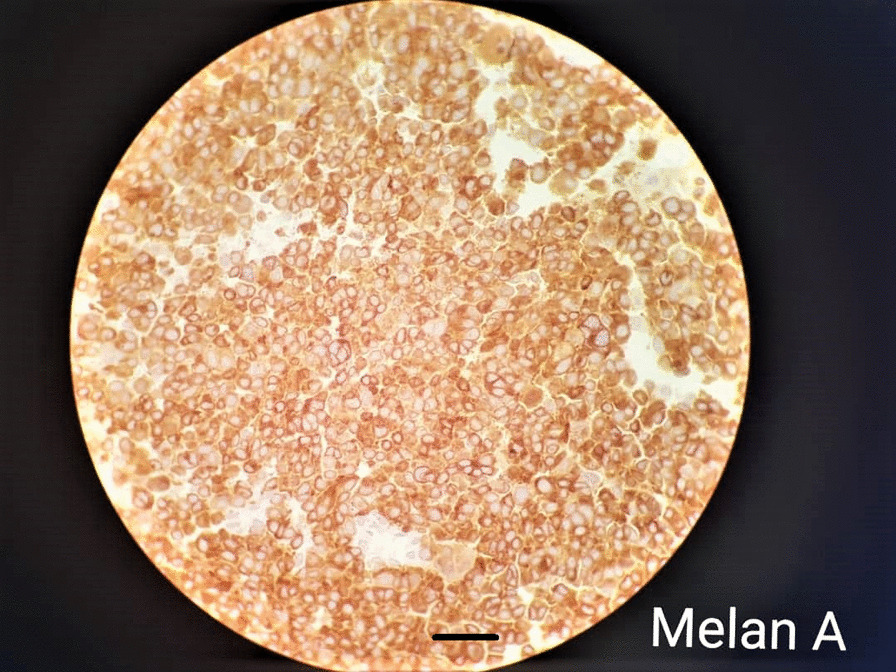
Immunohistochemical Melan-A staining yielding positive results and indicating melanoma (magnification 40 × 10, 30 μm scale bar, Olympus BX51 microscope, Nikon E400 camera)

The H&E-stained specimens of submandibular sections demonstrated lymphoid tissue associated with a malignant neoplasm composed of sheets of highly atypical cells with pleomorphic large vesicular, prominent nuclei (Fig. 6). Extensive necrosis and the perivascular rosette could also be seen. Furthermore, result from immunohistochemical (IHC) analysis yielded positive for HMB45 which further cemented the definitive diagnosis of melanoma (Fig. 7). Further IHC staining results are as follows: Pan CK (negative), Vimentin (positive), CD20 (negative in malignant cells), LCA (negative in malignant cells), Melan A (positive in malignant cells), Ki67 (60% proliferation rate) (Figs. 8, 9).

Hematoxylin and eosin stain (H&E) sections of left maxillary gingival mucosa demonstrated an oral melanoacanthoma which was irrelevant to the melanoma.

### Follow up

The patient’s 12-month follow up did not show any signs of recurrence.

## Discussion and conclusion

Primary melanomas of salivary glands have been all but one described to be arising in the parotid gland, most of which being the secondary metastasis of a distant cutaneous melanoma of head and neck region [10]. Furthermore, since melanocytic cells are not embryologically a component of the salivary glands, the detection and diagnosis of primary melanoma in a salivary gland remains an area of debate since anatomically lymph nodes have not been described for the submandibular salivary gland. Therefore, reaching a definitive diagnosis whether the melanoma has been formed de novo or is formed as a result of a metastasis from a distant origin is quite challenging in such cases [11]. However, Greene and Bernier [12] argue that downgrowth of the oral epithelium into the salivary glands can lead to decent and probable presence of melanoblastic cells within salivary glands, hence yielding the occurrence of primary malignant melanoma within salivary glands theoretically plausible; although, metastatic seeding of melanoma from a distant origin seems to be more probable. Metastatic seeding of malignant melanoma from unknown primary sources make up 3% of all diagnosed cases of melanoma; two thirds of which being established in lymph nodes [13]. These occurrences can be attributed to a number of factors such as a formerly excised lesion with misdiagnosis of melanoma or a spontaneous recurrences of a primary melanoma which can be seen in 10–35% of melanoma cases [14, 15]. Hence, it further emphasizes the role of obtaining thorough and comprehensive medical history and carrying out meticulous physical examinations in order to detect other possible cutaneous melanomas.

There is a dearth of evidence surrounding the primary melanomas arising from salivary glands, and submandibular glands in particular. Cochrane and Kenny [16] described a case of melanoma occurring within a pleomorphic adenoma of a submandibular gland. Unlike to this case, the patient had multiple melanotic lesions on his body, suggesting that the melanoma might have been a metastasis from a distant cutaneous source. In another case described by Agarwal et al. [17] a submandibular gland was operated on with suspicion of a neoplasm, which further was revealed to be a metastatic melanoma of the overlying skin of the submandibular region and the gland itself happened to be free of any metastasis. To date, there has only been one similar case described by Gopala Krishnan et al. [11] who identified a primary malignant melanoma of the submandibular gland.

Immunohistochemical (IHC) analysis utilizing markers such as HMB45, SOX-10, S-100, MITF, and MART-1 remain the most accurate measures for establishing a definitive diagnosis of melanoma, since histopathological analysis cannot always be relied on due the similarity of histological manifestations of melanoma to other lesions [18].

This report emphasizes the importance of thorough examination and prompt referral to designated specialists in cases with suspicious behavior which are unresponsive to treatments. It can be further concluded that melanoma can mimic a range of benign pathologies; therefore, putting it in the list of differential diagnosis of similar lesions seems plausible.

## Data Availability

Data sharing is not applicable to this article as no datasets were generated or analysed during the current study.

## Abbreviations

MM: Malignant melanoma
WHO: World Health Organization
CBCT: Cone-beam computed tomography
SAD: Short-axis diameter
MBq: Megabecquerel
^99m^Tc-MDP: Technetium 99 m-methyl diphosphonate
MRI: Magnetic resonance imaging
IHC: Immunohistochemical
H&E: Hematoxylin and eosin
HMB45: Human Melanoma Black-45
SOX-10: SRY-related HMG-box-10
MITF: Micropthalmia transcription factor
MART-1: Melanocyte Antigen Related to T-cells-1
Pan CK: Pan-Cytokeratin
LCA: Leukocyte common antigen
